# Arthritis and associated limitations in community-dwelling Canadians living with stroke

**DOI:** 10.1186/s12883-016-0636-x

**Published:** 2016-07-26

**Authors:** Kara K. Patterson, Kathryn M. Sibley

**Affiliations:** 1 Department of Physical Therapy, University of Toronto, 160-500 University Avenue, Toronto, ON M5G 1V7 Canada; 2 Toronto Rehab, University Health Network, Toronto, ON Canada; 3 Department of Community Health Sciences, University of Manitoba, Winnipeg, MB Canada; 4 Centre for Healthcare Innovation, Winnipeg, MB Canada

**Keywords:** Stroke, Arthritis, Limitations, Community

## Abstract

**Background:**

Residual impairments and gait deviations post-stroke may lead to secondary musculoskeletal complications such as arthritis. This study explored the prevalence of arthritis and associated functional limitations in community-dwelling Canadians with and without stroke.

**Methods:**

Secondary analysis of the Canadian Community Health Survey; a population-based, cross-sectional survey conducted by Statistics Canada in 2011 and 2012. Respondents >50 years old who reported a stroke diagnosis (*n* = 1892) were age- and gender-matched with controls randomly selected from survey respondents who did not report a stroke (*n* = 1892). Stroke and control groups were compared on presence of arthritis (yes/no) and secondary variables including pain, perceived health and assistance required (5 point scales) using the Rao-Scott X^2^ test. Within the stroke group, logistic regression was used to investigate the effect of arthritis on life satisfaction, pain limiting activities and perceived health with age, gender, BMI, comorbidities and socioeconomic status used as covariates in the model.

**Results:**

A greater proportion of the stroke group (53 %) reported arthritis compared to controls (43 %). These groups also differed in reports of perceived health and pain. Within the stroke group, those with arthritis were significantly more likely to report pain limiting activities (OR 3.89) and less likely to report satisfaction with life (OR 0.59).

**Conclusions:**

This preliminary work suggests that arthritis is more prevalent in individuals with stroke compared to individuals without stroke and that this co-morbidity is associated with worse reports of pain and perceived health. A limitation is that it is not possible to determine if the arthritis pre-dated or followed the stroke. This work provides support for a longitudinal investigation of the development of secondary musculoskeletal issues post-stroke.

## Background

Stroke is a serious Canadian health issue with approximately 350 000 people living with the effects of stroke [1]. Stroke-related impairments can cause walking dysfunction such as decreased velocity and spatiotemporal asymmetry, which can limit independence and quality of life [2, 3]. This walking dysfunction persists long after stroke rehabilitation is complete [4]. Improving mobility and increasing community ambulation after discharge from rehabilitation are common foci of current research [5, 6]. What has received far less attention, but may be of equal importance is the development of secondary musculoskeletal (MSK) complications as a result of persisting stroke-related gait deviations.

The link between gait deviations and MSK complications has been demonstrated previously. For example, MSK issues are prevalent in the amputee population with 40 % of people with unilateral amputation reporting pain in their intact limb compared to 20 % of people without amputation [7]. Furthermore, the prevalence of knee osteoarthritis (OA) among people with amputation is greater; 16 % report OA compared to only 11.7 % of people without amputation [7]. This increased prevalence of OA and pain in the amputee population has been attributed to their temporally asymmetric gait pattern [7, 8]. The increased stance duration exposes the intact limb to excessive, repetitive loading. This abnormal loading is commonly accepted as one mechanism that leads to pain and degeneration at the knee and increased risk of developing knee OA [9–11].

Post-stroke gait features the same temporally asymmetric gait pattern, with 55 % of individuals with chronic stroke exhibiting increased stance duration on their non-paretic limb compared to their paretic limb [4]. However, in comparison to the work with the amputee population, little has been done to investigate secondary MSK complications post-stroke. Lower limb pain is an issue post-stroke and the problem may get worse over time. Thirty five percent of individuals with stroke report pain in their lower limbs and the prevalence did not change over a 16 month period at which point 39 % of individuals reported lower limb pain [12]. This was not a statistically significant increase. But, given that intervals greater than 2 years may be needed to detect changes in joint cartilage secondary to gait deviations [9], it is possible that the prevalence of lower limb pain may have increased in the aforementioned study, if the participants were followed over a longer period of time. The potential for the development of secondary MSK issues, such as OA, long after discharge from stroke rehabilitation, is of great concern. This concern is not only because of the relationship between pain, limitations in activities of daily life and quality of life that has already been established in individuals with OA [13, 14]; but also because secondary MSK issues could exacerbate post-stroke gait dysfunction which could further restrict independence and limit quality of life. An important preliminary step to prepare for longitudinal examination of MSK complications after stroke is to develop an understanding for the prevalence and nature of MSK complaints, such as pain and arthritis, in the stroke population.

The primary objective of this study was to determine the prevalence of arthritis in adult Canadians living in the community with stroke compared to the prevalence of arthritis in those without stroke. The secondary objective was to understand the impact of stroke combined with arthritis on reports of pain, independence, perceived health and life satisfaction compared to the impact of stroke alone.

## Methods

A secondary data analysis was conducted using 2011 and 2012 annual components of the Canadian Community Health Survey (CCHS). The CCHS was a population-based, cross-sectional survey conducted by Statistics Canada between January 2011 and December 2012. Details regarding the survey, (e.g. data sources, methodology, questionnaire, data accuracy) are described online [15, 16] and in a published report [17]. In brief, a multistage stratified cluster design sampling procedure based on the Canadian Labour Force Survey (LFS) was used, and included people aged 12 and over living in private dwellings in all provinces and territories. Individuals were excluded if they lived on indigenous reserves or Crown Lands, institutions, certain remote regions, or were full-time members of the Canadian Forces. The CCHS covers approximately 98 % of the Canadian population aged 12 and over. Information was collected about diseases and health conditions, health, health care services, lifestyle and social conditions, mental health and well-being, and prevention and detection of disease. The response rate was 68.4 % with 126, 645 individuals completing the survey. Multiple quality control steps were used, including error detection by the computer-assisted interview software, validation with common outcomes from other Canadian population surveys, and external validation by federal and provincial partners.

All statistical analyses were performed with SAS 9.3 software (SAS Institute Inc., Cary, NC). The current study includes data from 1892 individuals aged 50 years old and older, who answered yes to the question, “have you been diagnosed with a stroke?” Of the remaining individuals who answered no to this question, age- and gender-matched individuals were randomly selected from the CCHS database to create a control group for comparison.

Data for the present study were derived from questions throughout the survey and were collapsed or re-categorized where appropriate. The primary outcome for this study was the presence of arthritis (binary: yes/no) derived from the response to the CCHS survey question “Do you have arthritis, excluding fibromyalgia?” Secondary outcome variables related to the impact of stroke and arthritis included: pain limiting activities, perceived health, life satisfaction and need for assistance with daily tasks. Other relevant descriptors that were related either to the secondary outcome variables and/or the risk for stroke and arthritis were: age, gender, body mass index (BMI), education, income, history of smoking, and presence of and number of comorbidities. Since a gold standard index of chronic disease does not exist, comorbidities included in the present study were selected according to a recommendation of 8 conditions based on the prevalence as a representative standard [18]. These included cancer, hypertension, diabetes, depression, heart disease, and chronic obstructive pulmonary disease (COPD), stroke and arthritis. Since stroke and arthritis were conditions already of interest in the present study, the presence of the remaining 6 conditions as well as the total number of comorbidities (with a maximum value of 6) were extracted. Descriptive statistics were calculated for all variables. Proportional differences in demographics, chronic conditions and outcome variables between groups were compared with the Rao-Scott X^2^ test.

### Comparisons between stroke and control groups

In order to address the primary research objective (prevalence of arthritis in individuals with stroke) the stroke and control groups were compared on the primary variable: presence of arthritis. The two groups were also compared on age, gender, BMI, income, education, history of smoking and presence of comorbidities. To further describe the differences between these two groups, we also investigated differences in the report of perceived general health and pain as it related to restriction of activities.

### Comparison within the stroke group of individuals with and without arthritis

In order to address the secondary objective (impact of arthritis on individuals with stroke), two analyses were conducted. First, comparisons were made between individuals with stroke and arthritis (stroke + arthritis) and individuals with stroke only on demographics, comorbidities and secondary variables of interest. Next, logistic regression was used to assess the impact of arthritis on pain, life satisfaction, perceived health and assistance with tasks. These outcome variables were first collapsed into binary responses. Report of pain with respect to limitation of activities was collapsed to pain restricts activities (yes/no), rating of perceived health was collapsed to satisfactory (yes/no) and rating of life sa**t**isfaction was collapsed to satisfied (yes/no) and the need for assistance with daily activities was collapsed to needs assistance (yes/no). Second, binary logistic regressions modelled pain, perceived health, life satisfaction and assistance with daily activities in the individuals with stroke as a function of the presence of arthritis. However, there were too few observations to carry out a logistic regression (*n* = 23) for assistance with daily activities. Third, in the case of a significant effect of arthritis, the logistic regression model was adjusted to include age, gender, BMI, education, income, history of smoking and number of comorbidities (max. 6) as covariates. Odds ratios with 95 % confidence intervals (CI) were reported for significant associations.

## Results

### Comparison of stroke and control groups

**Fig. 1:**
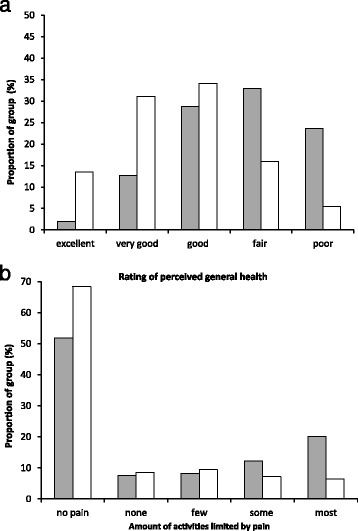
Comparison of individuals with and without stroke. The proportion of individuals in the stroke (*shaded bars*) and control group (*open bars*) reporting on **a** general health and **b** pain related to how it limits activities. The proportions of responses for both questions were significantly different between the groups (*p* < 0.0001)

The total number of individuals selected from the CCHS database was *n* = 3784. A greater proportion of the stroke group, 53 % (*n* = 1010) compared to the control group 43 % (*n* = 812) reported having arthritis (*p* < 0.0001). Table 1 summarizes the characteristics of the stroke and control groups with respect to age, gender, BMI, education, income, history of smoking and comorbidities. Due to age- and gender- matching, the mean age and gender distribution was the same between the stroke and control groups. The stroke and control groups were significantly different in education (*p* < 0.0001), income (*p* < 0.0001), history of smoking (*p* < 0.0001) and the presence of all 6 comorbidities (p-values ranged from *p* < 0.0001 to *p* = 0.02). The responses of individuals in the stroke and control groups reporting on perceived health and pain related to the amount of activities it prevents are illustrated in Fig. 1. The proportions of responses to questions related to general health (*p* < 0.0001) and pain (*p* < 0.0001) were different between the stroke and control groups.

**Table 1:** Demographics of survey respondents with and without stroke and respondents with stroke with and without arthritis. Values are in percentage of responses

	Adults no stroke (*n* = 1892)	Adults with stroke (*n* = 1892)	Adults with stroke and arthritis (*n* = 1010)	Adults with stroke without arthritis (*n* = 882)
Gender^b^				
Male/Female	48/52	48/52	39/61	59/42
Age^b^				
50–54 years	4.4	4.4	3.6	5.3
55–59 years	8.1	8.1	7.3	9.0
60–64 years	13.1	13.1	11.5	15.0
65–69 years	14.1	14.1	13.2	15.2
70–74 years	15.2	15.2	15.0	15.5
75–79 years	16.2	16.2	16.7	15.5
80 + years	28.9	28.9	32.8	24.5
BMI^b^				
Underweight	1.8	2.8	2.9	2.6
Normal weight	38.3	37.4	32.8	42.8
Overweight	38.0	35.7	37.3	34.0
Obsese	21.9	24.1	27.0	20.7
History of smoking^a^				
Smoker (yes/no)	11.2/88.8	15.5/84.5	16.4/83.6	14.6/85.4
Education^a^				
< secondary school	31.2	37.9	39.7	39.0
Graduated secondary school	18.3	15.3	14.9	17.1
Some post-secondary	4.5	3.2	2.9	4.0
Graduate post-secondary	46.0	43.6	42.5	48.7
Income^ab^				
>$20, 000	15.4	25.1	27.4	22.4
$20,000 to $39,999	34.8	39.2	42.2	35.8
$40,000 to $59,999	22.0	17.8	16.2	19.6
$60,000 to $79,999	12.5	8.1	6.4	10.0
>$80,000	15.2	9.8	7.7	12.3
Comorbidities (yes/no)				
Cancer^a^	6.5/93.5	8.5/91.5	9.2/90.8	7.8/92.2
Hypertension^a^	44.0/56.0	63.0/37.0	64.8/35.2	60.8/39.2
Diabetes^a^	15.3/84.7	26.2/73.8	28.0/72.0	24.2/75.8
Depression^a^	6.8/93.2	21.3/78.7	19.9/80.1	23.0/77.0
Heart disease^ab^	16.4/83.6	39.7/60.3	43.6/56.4	35.3/64.7
COPD^ab^	8.7/91.3	14.3/85.7	19.4/80.6	8.4/91.6

*Abbreviations: COPD* chronic obstructive pulmonary disease

^a^significant difference between adults with and without stroke (*p* < 0.0001)

^b^significant difference between individuals with stroke and arthritis and individuals with stroke only (*p* < 0.01)

### Comparison within the stroke group of individuals with and without arthritis

Table 1 summarizes the characteristics of the stroke group of individuals with and without arthritis. Within the stroke group, those with arthritis were significantly different from those without arthritis on several demographics; gender (*p* < 0.0001), age (*p* = 0.001), BMI (*p* = 0.0004) and income (*p* < 0.0001) as well as the presence of two comorbidities; heart disease (*p* = 0.0003) and COPD (*p* < 0.0001).

**Fig. 2:**
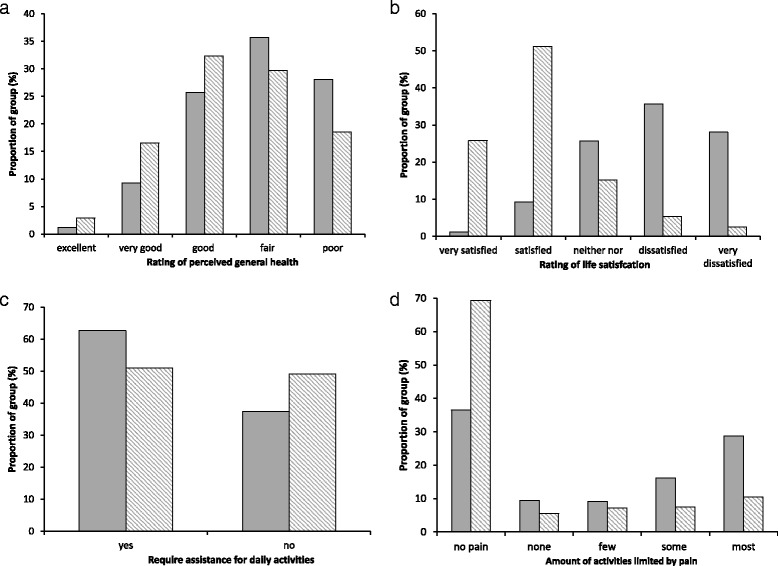
Comparison of individuals with stroke and arthritis and those with stroke only. The proportion of individuals with stroke and arthritis (*shaded bars*) and stroke only group (*hatched bars*) reporting on **a** general health, **b** life satisfaction, **c** need for assistance with daily activities and **d** pain related to how it limits activities. The proportions of responses for both questions were significantly different between the groups (*p* < 0.0001, *p* < 0.0001, *p* = 0.0016, *p* < 0.0001 respectively)

The impact of stroke with arthritis compared to stroke alone on the secondary variables of interest is illustrated Fig. 2. The proportions of responses to questions related to perceived health (*p* < 0.0001), pain (*p* < 0.0001), life satisfaction (*p* < 0.0001), and required assistance (*p* = 0.002) were different in the stroke + arthritis group compared to the stroke alone group. The results for the logistic regression analyses are summarized in Table 2. In individuals with stroke, arthritis had a significant effect on pain (*p* < 0.0001) and life satisfaction (*p* < 0.0001) but not perceived health (*p* = 0.19) when age, gender, BMI, income, education, history of smoking, and number of comorbidities are accounted for.

**Table 2:** Logistic regression. The effect of arthritis in individuals with stroke

	X^2^	p-value	Odds ratio
Pain	132.75	<0.0001	3.89
Life satisfaction	16.72	<0.0001	0.59
Perceived health	1.75	0.19	n/a

*Abbreviations: n/a* not applicable as relationship was not significant

## Discussion

The main finding of this study is that the prevalence of arthritis in community-dwelling adults is greater in those with stroke (53 %) compared to those without a stroke (43 %) of similar age and gender. Similar to the present study, a cross-sectional study in the amputee population suggested a link between disease-induced gait deviations and arthritis; individuals with amputation had a prevalence ratio of 2.2 for knee pain and 1.5 for knee OA compared to individuals without amputation.

The individuals with stroke were also different in terms of education and income and more likely to report a history of smoking, and the presence of comorbidities compared to the group without stroke. The increased report of smoking is not surprising given that smoking is a risk factor for stroke [19]. The group differences in education and income are consistent with the inverse relationship between socioeconomic status (SES) and the incidence of stroke reported in previous work [20]. Finally, the greater prevalence of various chronic conditions in the stroke group is also expected given that the presence of one or more additional morbidities is 5.18 times more likely in individuals with stroke compared to those without stroke [21].

To our knowledge, this is the first study that examined the prevalence of arthritis in community-dwelling individuals with stroke. A related study that analyzed CCHS data from 2010, found that individuals with arthritis have higher odds of stroke [22]. Both studies share the same limitation; given the cross-sectional nature of the data, it is impossible to determine if the arthritis preceded or followed the stroke. However, the present results combined with this previous analysis of CCHS data [22], suggests a relationship between stroke and arthritis that is both complex and potentially bidirectional. In individuals with arthritis, an elevated rate of stroke may be attributed to multiple factors including chronic inflammation, long-term use of glucocorticoids and reduced physical activity limited by pain and mobility impairments [22]. Conversely, individuals with stroke may be more likely than individuals without stroke to develop arthritis as a secondary complication related to abnormal loading associated with stroke-induced gait deviations. Thus, a prospective, longitudinal investigation of the direction of the relationship between stroke and arthritis seems warranted.

The present results also revealed key differences between the stroke + arthritis group and the stroke alone group. First, there were more women in the stroke + arthritis group which can be attributed to the fact that in general, women are at greater risk to develop OA compared to men [23]. Second, the stroke + arthritis group was older and heavier than the stroke alone group. The cross-sectional nature of the analysis precludes any conclusions about the direction of this relationship. It is possible that those in the stroke + arthritis group exhibit arthritis because they had been exposed longer to the abnormal joint load imposed by the primary stroke-related motor impairments and this led to decreased mobility which in turn led to weight gain and increased BMI. Alternatively, it is possible that individuals with stroke who are older and have larger BMI values are at greater risk for developing arthritis compared to those with stroke alone. The stroke + arthritis group was also different in reported income and a greater proportion of the stroke + arthritis group had heart disease and COPD than the stroke only group. Finally, the combination of stroke and arthritis appears to have a negative impact on perceived health, life satisfaction, independence and pain. When other contributing factors such as SES and comorbidity are considered, individuals with stroke and arthritis are significantly more likely (OR = 3.89, 95 % CI 3.09, 4.90) to report pain that limits activity and less likely (OR = 0.59, 95 % CI 0.46, 0.76) to report satisfaction with life. Inferences about casual relationships are not possible, however, the data do indicate a complex interaction of SES, multimorbidity and the impact of arthritis on the independence and health of community-dwelling Canadians living with stroke. There is a link between SES and stroke severity [20] and previous work in the same population (i.e. Canadians with stroke) has indicated that, those with a lower SES are less likely to be treated by a neurologist and less likely to receive rehabilitation [24]. It is possible that this inequality of care leads to limited post-stroke recovery in individuals with lower SES which in turn leaves these individuals with greater motor deficits and gait deviations, thus increasing the risk of developing secondary complications such as arthritis.

As is the case with all secondary analyses, this study has a few limitations. First, the analysis is based on self-report data and thus, is subject to healthy responder and recall bias. Second, the dataset included community-dwelling adults which may restrict the generalizability of our findings to higher functioning individuals potentially with less severe stroke. Finally, due to the nature of the questions in the original CCHS survey, it is impossible to determine specific details regarding the arthritis including the joints affected, whether the paretic or non-paretic limb was affected and whether the arthritis preceded or followed the stroke. However, the increased prevalence of arthritis among individuals with stroke lends support to the hypotheses that stroke-related gait deviations may lead to secondary MSK complications. It is possible that given the temporally asymmetric post-stroke gait pattern, which is similar to the gait pattern in the amputee population, at least one contributing factor to the increased prevalence of arthritis is related to abnormal loading of the lower extremities. These results suggest that it may be worthwhile to conduct a longitudinal study of secondary MSK complaints post-stroke.

## Conclusions

In conclusion, community-dwelling individuals with stroke are more likely to report arthritis compared to age- and gender-matched adults without stroke and the combination of stroke and arthritis increases the likelihood of pain limiting activities and decreases the likelihood of satisfaction with life. Future work should investigate longitudinally, the potential development of secondary complications like arthritis and joint pain and the negative impact on health, quality of life and independence post-stroke.

## Abbreviations

BMI, body mass index; CCHS, Canadian Community Health Survey; MSK, musculoskeletal; OA, osteoarthritis

## References

[1] Hakim AM, Silver F, Hodgson C (1998). Organized stroke care: A new era in stroke prevention and treatment. Can Med Assoc J.

[2] Schmid A, Duncan PW, Studenski S, Lai SM, Richards L, Perera S (2007). Improvements in speed-based gait classifications are meaningful. Stroke.

[3] Lewek MD, Bradley CE, Wutzke CJ, Zinder SM (2013). The relationship between spatiotemporal gait asymmetry and balance in individuals with chronic stroke. J Appl Biomech.

[4] Patterson KK, Parafianowicz I, Danells CJ, Closson V, Verrier MC, Staines WR (2008). Gait asymmetry in community-ambulating stroke survivors. Arch Phys Med Rehabil.

[5] Lord SE, Rochester L (2005). Measurement of community ambulation after stroke: current status and future developments. Stroke.

[6] Lord S, McPherson KM, McNaughton HK, Rochester L, Weatherall M (2008). How feasible is the attainment of community ambulation after stroke? A pilot randomized controlled trial to evaluate community-based physiotherapy in subacute stroke. Clin Rehabil.

[7] Norvell DC, Czerniecki JM, Reiber GE, Maynard C, Pecoraro JA, Weiss NS (2005). The prevalence of knee pain and symptomatic knee osteoarthritis among veteran traumatic amputees and nonamputees. Arch Phys Med Rehabil.

[8] Nolan L, Wit A, Dudzinski K, Lees A, Lake M, Wychowanski M (2003). Adjustments in gait symmetry with walking speed in trans-femoral and trans-tibial amputees. Gait Posture.

[9] Maly MR (2008). Abnormal and cumulative loading in knee osteoarthritis. Curr Opin Rheumatol.

[10] Andriacchi T (2006). Mundermann: The role of ambulatory mechanics in the initiation and progression of knee osteoarthritis. Curr Opin Rheumatol.

[11] Miyazaki T, Wada M, Kawahara H, Sato M, Baba H, Shimada S (2002). Dynamic load at baseline can predict radiographic disease progression in medial compartment knee osteoarthritis. Ann Rheum Dis.

[12] Jonsson AC, Lindgren I, Hallstrom B, Norrving B, Lindgren A (2006). Prevalence and intensity of pain after stroke: a population based study focusing on patients’ perspectives. J Neurol Neurosurg Psychiatry.

[13] Jordan J, Luta G, Renner J, Dragomir A, Hochberg M, Fryer J (1997). Knee pain and knee osteoarthritis severity in self-reported task specific disability: the Johnston county osteoarthritis project. J Rheumatol.

[14] Salaffi F, Carotti M, Stancati A, Grassi W (2005). Health-related quality of life in older adults with symptomatic hip and knee osteoarthritis: a comparison with matched healthy controls. Aging Clin Exp Res.

[15] Statistics CanadaCanadian Community Health Survey - Annual Component (CCHS). 2011. http://www23.statcan.gc.ca/imdb/p2SV.pl?Function=getSurvey&Id=114112 Accessed 6 July 2016.

[16] Statistics CanadaCanadian Community Health Survey - Annual Component (CCHS). 2012. http://www23.statcan.gc.ca/imdb/p2SV.pl?Function=getSurvey&Id=13592 Accessed 6 July 2016.

[17] Statistics Canada (2013). Canadian Community Health Survey (CCHS) annual component user guide 2012 and 2011-2012 microdata files.

[18] Diederichs C, Berger K, Bartels DB (2011). The measurement of multiple chronic diseases: a systematic review on existing multimorbidity indices. The Journals of Gerontology Series A.

[19] Shinton R, Beevers G (1989). Meta-analysis of relation between cigarette smoking and stroke. Br Med J.

[20] Cox AM, McKevitt C, Rudd AG, Wolfe CDA (2006). Socioeconomic status and stroke. The Lancet Neurology.

[21] Gallacher KI, Batty GD, McLean G, Mercer SW, Guthrie B, May CR (2014). Stroke, multimorbidity and polypharmacy in a nationally representative sample of 1,424,378 patients in Scotland: implications for treatment burden. BMC Med.

[22] Matveev R, Ardern CI. Co-occurrence of arthritis and stroke amongst middle-aged and older adults in Canada. Stroke research and treatment. 2014;2014. 10.1155/2014/651921 PMC4009210 24834358

[23] Manninen P, Riihimaki H, Heliovaara M, Makela P (1996). Overweight, gender and knee osteoarthritis. Int J Obes Relat Metab Disord.

[24] Kapral MK, Wang H, Mamdani M, Tu JV (2002). Effect of socioeconomic status on treatment and mortality after stroke. Stroke.

[25] Statistics CanadaCanadian Community Health Survey: Public Use Microdata File, 2011/2012.2013.http://www5.statcan.gc.ca/olc-cel/olc.action?ObjId=82M0013X2013001&ObjType=46&lang=en. Accessed 15 April 2016.

